# Why do Social Workers Leave? A Moderated Mediation of Professionalism, Job Satisfaction, and Managerialism

**DOI:** 10.3390/ijerph20010230

**Published:** 2022-12-23

**Authors:** Ziyu Liu, Hung Wong, Jifang Liu

**Affiliations:** 1 Department of Social Work, Chinese University of Hong Kong, Hong Kong, China; 2 School of Finance, Jilin University of Finance and Economics, Changchun 130117, China

**Keywords:** social work, professionalism, job satisfaction, turnover intention, managerialism

## Abstract

Turnover has been a serious concern to social service organizations. A lack of committed social workers is a risk to organizational performance and service quality. Therefore, it is vital to better understand the leaving process of social work practitioners. The study constructed a moderated mediation model to examine the mediating role of job satisfaction between employees’ professionalism and turnover intention and the moderating role of the perceived level of managerialism in the context of social work organizations. A total of 667 participants from Guangzhou, Shenzhen, and Shanghai in China were recruited to complete the survey. Results presented that job satisfaction plays a full mediation role in the relationship between professionalism and turnover intention. In addition, the positive relationship between professionalism and job satisfaction, as well as the negative relationship between professionalism and turnover intention were moderated by managerialism. The findings enrich knowledge about turnover among social workers in the context of China and inspire to foster professionalism among service workers to improve job satisfaction and alleviate turnover intention and actual turnover as well as to apply management techniques and structures properly to strengthen the effect of professionalism on promoting job satisfaction and on preventing turnover intention.

## 1. Introduction

Turnover of employees in social service agencies is a serious concern [1]. In China, social worker turnover has been a major concern in professional development. In 2014, the social worker turnover rate in Shenzhen reached 22.2%, exceeding the 20% alarm line of the industry [2]. In 2017, 14% of social workers in Dongguan had either left their currently employed organization or left the field [3]. In 2022, Shanghai reported that the turnover rate had exceeded 20% for community social workers [4]. The high rates of turnover in the social work field have severe implications for the quality, consistency, and stability of services provided to clients in need [5,6]. Meanwhile, in the context of China, as one of the means to innovate social governance, social work is playing an increasingly important role in assisting governments in service provision [7]. Accordingly, effectively retaining workers is a crucial task for both social work organizations and the government [8].

The professionalism of employees can provide an important mechanism for reducing turnover theoretically [9]. Professionalism incorporates attitudes representing levels of identification with and commitment to a particular profession [10]. Avolio et al. [11] found that social and personal identification affect employee behavior through their work attitudes. In nursing, professionalism is identified as a fundamental concept, which plays a vital role in enhancing job satisfaction, retaining nurses, and improving clinical outcomes for patients [12]. However, research on professionalism in the social work literature has been surprisingly scarce. In addition, in the context of the vast government procurement of social services in China, the focus of social service is shifting rapidly to a more business-oriented model. However, the influence of such managerialism on social workers’ professionalism remains unclear.

This study offers several knowledge contributions and practical implications. First, we extend the turnover literature by presenting the role of professionalism in the employee’s leaving process. This is the first time in the social work field that professionalism has been examined and identified regarding its role in professionals’ attitudes and behaviors. Second, by presenting managerialism as a new moderator of professionalism, we respond to a gap in the literature concerning the role management notions, techniques, and structures will play in affecting professionalism. The current study provides a novel theoretical explanation in explaining why social service workers leave or stay. 

The goal of the present study is twofold. First, we set out to examine the underlying process through which professionalism affects social workers’ turnover intention by focusing on job satisfaction. In most turnover studies, turnover intention was used as an outcome variable since the intention to leave is the single strongest predictor of actual turnover [13,14]. Moreover, it is more practical to track employees’ intention to quit in a cross-sectional study than track their actual turnover through a longitudinal study. Therefore, the present study focuses on examining employees’ turnover intention through which we are able to predict their actual turnover. Second, we explore the moderating role of managerialism on the hypothesized mediation model. Theoretical contributions and practical suggestions are presented to reduce social workers’ intent to quit and eventually reduce the actual turnover rates.

## 2. Theoretical Model and Hypotheses

**Figure 1:**
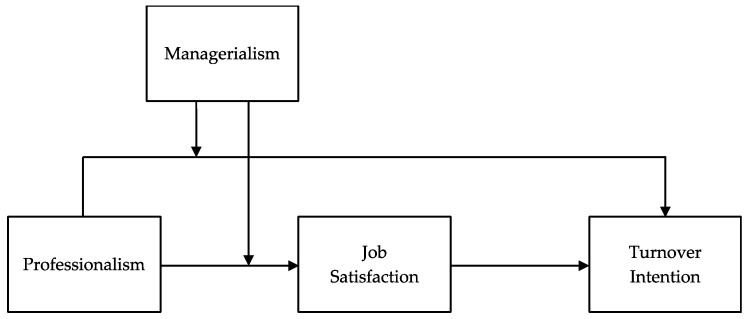
Hypothesized mediated moderation model.

Our proposed theoretical model is depicted in Figure 1. As the value orientation and standards of professionals, professionalism reflects work attitude and concepts of the certain profession [15]. Wynd [10] defined professionalism as attitudes representing levels of identification with a particular profession. Social identity theory proposes that employees’ organizational identification—knowledge that he or she belongs to an organization—can lead to positive work attitudes such as job satisfaction [16] and is negatively related to withdrawal intention and the following behaviors [17]. In addition, in professional organizations, the notion of managerialism has been long considered a factor having an impact on professionalism [18]. 

### 2.1. Professionalism and Turnover Intention

Turnover intention was conceived to be a conscious and deliberate willfulness to leave the organization [19]. It is described as the last in a sequence of withdrawal cognitions, and the strongest cognitive precursor of actual turnover [20]. High turnover has been recognized as a major problem in public welfare agencies and human service organizations because it impedes the effective and efficient delivery of service and disrupts the quality of care to those needing services [21]. Turnover intention antecedents are various. Human service workers like teachers and social workers who have demanding jobs are often under high pressure and tend to experience mental health issues such as anxiety, distress, and burnout, which may lead to the intention to quit [22,23,24]. In Barak and colleagues’ [21] meta-analytical study of turnover intention among human service employees, they found that burnout, job dissatisfaction, low organizational and professional commitment, stress, and lack of social support are the strongest predictors of turnover intention, suggesting that the major predictors of leaving are job-based, but not personal or related to the balance between work and family. Other job-based factors that lead to employees’ intention to leave have also been identified, such as perceived discrimination at work [25] and fairness-management practice [5]. 

Examining the relevant literature suggests a link between professionalism and staff absenteeism and turnover [9,26]. Improving professionalism has a positive impact on the willingness to continue in the profession [27]. In the field of social work, professionalism is a necessary quality of social work students and practitioners in order to improve career development and the quality of service [28]. Kim and Kao [29], in their meta-analysis of turnover intention predictors, demonstrated that professionalism, as a type of organizational culture, had a deterring effect on turnover intention among U.S. child welfare workers. In addition, social work educators usually regard the feeling of devotion to the profession, which comes from altruism, belief in public service as well as autonomy, as the primary aim of social work education [30,31]. Such dedication to the profession was reported by many social workers as a crucial reason to stay under the condition of low pay, few promotion opportunities, unclear career development, and low public identity [31,32]. Therefore, we propose the following hypothesis:

**Hypothesis** **1.** *Professionalism is negatively related to turnover intention*.

### 2.2. The Mediating Role of Job Satisfaction

The impact of professionalism on turnover intention could be mediated through employees’ level of job satisfaction [33]. Job satisfaction is considered to be a set of attitudes towards various aspects of the job, such as pay, workgroup, organizational factors, and work environment [34]. Employees who are satisfied with their jobs are more likely to keep working at an institution and less likely to quit [35,36]. Empirical studies provide consistent evidence that job satisfaction is a strong predictor of staff turnover intention as well as actual turnover behavior [37,38,39,40].

The attitudes and ideology held by its practitioners denote the degree of professionalism characteristics of an occupation [41]. These attitudinal attributes are considered theoretical dimensions of professionalism, which include autonomy, the use of professional organizations as major referents, and the identification with and commitment to the profession [42]. Herzberg’s theory [43,44] suggests that deficits in organizational factors such as pay, benefits, incentives, and supervision are related to dissatisfaction, while motivation variables such as achievement, commitment, identification, and autonomy are factors associated with job satisfaction. Therefore, professionalism may be a motivator associated with job satisfaction. Existing studies have examined the role of professionalism in affecting job satisfaction. For instance, Shen et al. [27] indicated that professionalism has a positive impact on job satisfaction and the willingness to continue in the profession. Celik and Hisar [26] suggested that nurse professionalism is positively related to job satisfaction. In addition, studies demonstrated that employees with lower levels of work commitment are less satisfied with their jobs and more likely to play to leave the organization [14,35,45,46]. Therefore, we propose the following hypothesis:

**Hypothesis** **2.** *Intrinsic job satisfaction mediates the negative relationship between professionalism and turnover intention*.

### 2.3. The Moderating Role of Managerialism

As a result of socio-economic and political developments such as budget constraints, accountability for quality, and decentralization of public service [47], Chinese social service organizations have adopted organizational strategies, structures, technologies, management instruments, and values that are commonly found in the private business sector [48]. This trend of copying techniques of the private sector by public organizations is one of the earliest features of managerialism and remains one of the most enduring [49].

Some researchers equate managerialism in the public sector with “good management” and suggest that the right proportion of managerialism may be needed in the field of human service due to rising expectations, shrinking budgets, and increased competition [50,51]. This is considered the “hybridity” perspective [52]. This judgment is challenged by others who argue that managerialism works against its own intentions of efficient and effective quality improvement [53,54,55]. This situation is considered a “resistance” thesis [56] or called a “managerialism contradiction” [57]. Such contradiction erodes professionals’ values that are in line with social work agency as a human service organization, that is characterized by professionalism, collegiality, and altruism [58,59]. On the one hand, the work of professionals in public welfare provision is notoriously difficult, as they are faced with challenges dealing with presumably conflicting (managerial and professional) requirements and objectives [60]. They are required to do a great deal of managerial work (e.g., planning, budgeting, performance appraisals, and management audits); they often feel more like alienated bureaucrats than professional practitioners in the organizational context [48]. On the other hand, professionals with administrative assignments must allegedly adjust their actions in response to a persistent dilemma of whether to serve “professional” interests in autonomy, collegiality, and service quality, or “managerial” interest in organizational productivity and economic efficiency [56]. A study by Smeenk et al. [57] on European university employees found that managerialism negatively affected normative commitment, while managerialism was positively related to continuance commitment. Tschannen-Moran [61] found that teachers’ professionalism is positively affected by the professional orientation of principals in their leadership style.

In summary, managerial values collide with professional values [62]. Professionals who worked in organizations that are more focused on efficiency as well as output and have more pressure from the bottom line are more likely to experience reduced professionalism [63,64]. Therefore, we propose the following hypotheses:

**Hypothesis** **3a.** *Managerialism moderates the relationship between professionalism and job satisfaction, such that the relationship is weaker for employees who perceived a high level of managerialism than those who perceived a low level of managerialism*.

**Hypothesis** **3b.** *Managerialism moderates the relationship between professionalism and turnover intention, such that the relationship is weaker for employees who perceived a high level of managerialism than those who perceived a low level of managerialism*.

## 3. Methods

### 3.1. Participants and Procedures

The study drew on a Web survey conducted from October 2021 to January 2022 among Chinese social workers sampled from 26 social work organizations over three cities in mainland China—Guangzhou, Shenzhen, and Shanghai. We chose these cities because they are expected to reflect different levels of professionalism and managerialism adoption [65], but at the same time are reasonably comparable in socio-economic terms. In addition, the number of well-established social work organizations in these cities is generally sufficient to be able to sample from.

Multistage cluster sampling was applied in the process of collecting data and carried out as follows. Firstly, 15 administrative districts were extracted from lists obtained from the Guangzhou, Shenzhen, and Shanghai governments (5 districts in each city). Secondly, in each district, a medium-to-large organization and a small organization were extracted based on lists of social work organizations obtained from governments’ social organization platforms. The inclusion criteria of medium-to-large social work organizations were those with more than 30 full-time workers, and those of small organizations were those employing fewer than 30 full-time social workers. All organizations had to be founded for at least one year and had direct services provided. At last, seven out of ten organizations in Guangzhou replied and agreed to attend the survey, eight organizations attended the survey in Shenzhen, but only two organizations in Shanghai replied and agreed to participate in the survey. In this sample organization selection, voluntary agreement was obtained from the leader of each organization. Along with the cooperation of the assigned contact person and the leaders of the participating organizations, 613 social workers from Guangzhou, 489 social workers from Shenzhen, and 66 social workers from Shanghai were invited to take part in this study (1168 in total).

Before conducting the survey, social workers were asked to confirm their willingness to participate. Respondents were asked to independently finish the online survey questionnaire by themselves. To ensure the authenticity and reliability of the questionnaire, participants were informed that the survey was anonymous, and the results will be used for solely academic purposes. A total of 826 social workers agreed to participate in the survey, resulting in a response rate of 70.7%. Among them, 667 were identified as valid responses. In terms of the demographic information of the participants, about 63% were less than 30 years old, 32% were between 30 and 40 years old, and approximately 5% were older than 40 years old; approximately 81% of the participants were female. The descriptive statistics of the sample are shown in Table 1.

### 3.2. Measures

Professionalism. Hall (1968) developed an attitude scale to measure the degree of professionalism among practitioners of various occupations. Snizek [41] modified Hall’s scale into a shortened version, named the Snizek-Revised Hall’s Professionalism Inventory Scale (C-SR-HPIS). In this study, professionalism was measured by 16 items on 5 dimensions adapted from Snizek’s [41] shortened version of the professionalism scale. The scale was validated in the Chinese context among clinical nurses [66], and thus, the scale had already been translated into Chinese. The dimension of the use of professional organizations as major referents (PO) included four items (e.g., “I systematically read the professional journals”); belief in public service (PS) included three items (e.g., “If ever an occupation was indispensable, it is social work”); belief in self-regulation (SR) contained three items (e.g., “My fellow professionals have a pretty good idea about each other’s competence”); sense of calling to the field (SC) contained four items (e.g., “Social workers have a real sense of ‘calling’ to their work”); and autonomy (AU) included two items (e.g., I make my own decisions in regard to what is to be done in my work”). All items were assessed on a 7-point Likert scale ranging from “1 = totally disagree” to “7 = totally agree.” The item scores of each dimension were averaged to create a mean score for the five dimensions respectively. The Cronbach’s alpha of the five subscales in this study were 0.849, 0.702, 0.705, 0.915, and 0.787, respectively.

Job satisfaction. Job satisfaction was measured using the four-item, five-point Likert scale developed and validated by Quinn and Staines [67]. The index measures an employee’s general affective reaction to the job without reference to any specific work facet [21]. The reliability of the scale in the current study was 0.84, indicating high internal consistency.

Turnover intention. Turnover intention was measured using five items adapted from Auerbach et al. [68] as well as Moynihan and Pandey [1], indicating social workers’ intentions to leave their organization. All items were assessed on a five-point Likert scale ranging from “1 = totally disagree” to “5 = totally agree.” Participants answered questions including “I once had the idea of leaving this organization”, “I cannot see any personal development in his organization”, “In the next few months I intend to leave this organization”, “In the few years I intend to leave this organization”, and “I would be very happy to spend the rest of my career with this organization”. The reliability of the scale in the current study was 0.83, indicating high internal consistency. 

Perceived level of managerialism. Managerial development was measured using a seven-item scale [69]. The scale measured the degree to which social work organizations were distinct from private sector organizations and how far managerial and professional discretion was fenced in by explicit standards and rules. The items involved the extent of segregation (expansion of service categories), competition between organizations, use of management practices drawn from the private sector, stress on discipline and frugality in resource use, emphasis on hands-on management, explicit and measurable standards of performance, and greater emphasis on output controls [69]. The participants were asked to indicate the extent to which they perceived these managerial developments as being applied to their organizations (1 = does not apply at all, 5 = applies completely). The reliability of the scale in the current study was 0.84, indicating high internal consistency.

Control variables. Previous studies pointed out that age, education, and tenure within the organization are significant predictors of turnover [21,70]. Specifically, younger and better-educated employees are more likely to leave than their counterparts [35,45]. In addition, turnover rates are significantly higher among employees with a shorter length of service than among those who are employed longer [71,72]. Therefore, this study used age, education, and years of work experience as control variables.

### 3.3. Data Analysis

Descriptive statistics (frequencies, percentages, means, and standard deviations) and preliminary analyses for the variables involved in this study were conducted in SPSS 24.0. Further, structural equation modeling (SEM) using maximum likelihood estimation was carried out using AMOS 24.0. Confirmatory factor analysis (CFA) was conducted to confirm the validity of each measurement used in the present study. In addition, in order to examine the mediation effect of job satisfaction on the association between professionalism and turnover intention, bootstrapping methods (5000 iterations for the present study) using 95% confidence intervals were employed. The effects are considered significant if there is no “zero” value between the upper and lower bounds of the confidence intervals [73]. Finally, the technique of hierarchical regression was used via SPSS 24.0 to test the potential moderating effects of managerialism. Hierarchical regression allows the direct assessment of change in explanatory power between interactive steps. Moreover, as a traditional technique, it provides a baseline set of results for the study’s predictions [74]. To reduce the potential effects of multicollinearity, all variables were standardized before conducting the regression.

## 4. Results

### 4.1. Common Method Biases

In order to avoid common method variance (CMV), Harman’s single-factor method was used for statistical control. The results showed that more than one factor was extracted with a characteristics value greater than 1, and the maximum degree to which a factor was explained was 32.284%, which was less than the 40% critical value [75]. Therefore, CMV was not a major threat in this sample.

### 4.2. Descriptive Statistics and Preliminary Analyses

The means, standard deviation, and correlation analysis of each variable in this study are shown in Table 2. Professionalism was significantly positively related to job satisfaction and significantly negatively associated with turnover intention. In addition, in order to examine the multivariate normality, skewness, and kurtosis were conducted and the values of all the variables in this study were within acceptable ranges of ±2 (range from −0.002 to 1.374) [76]. Moreover, the study examined variance inflation factors (VIF) to assess the likelihood that multicollinearity affects the results. The statistics of all the predictors (VIFs < 2.00) indicated that multicollinearity is not a concern in this study [77].

### 4.3. Confirmatory Factor Analysis

Confirmatory factor analysis was conducted using the maximum likelihood method of estimation and the results show a good fit index (χ2 = 313.58, df = 94, χ2/df = 3.34, CFI = 0.96, GFI = 0.95, TLI = 0.95, and RMSEA = 0.06). In addition, the standard factor loadings of the observable variably in this study are between 0.38 and 0.85, all above the acceptable loading of 0.30 [78]. Therefore, the results of the measurement model indicate that the selected observable variable efficiently reflects the essential structure of the latent variable in this study.

### 4.4. Test of Structural Model

The structural model indicated a good model fit to the data, Chi-square (χ2 = 298.78, df = 128, *p* < 0.001) was significant, NFI (0.93), IFI (0.96), and CFI (0.96) were all larger than the cut-off value of 0.90, and RMSEA (0.05) was less than the acceptable value of 0.08. Furthermore, the results showed that the whole model can explain 38% of social workers’ intention to quit their job.

**Figure 2:**
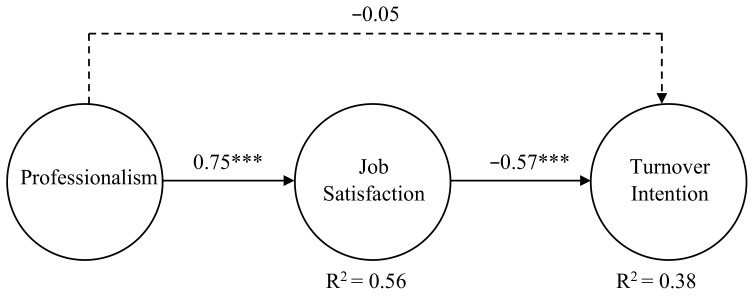
Overall model. Note: *** *p* < 0.001.

The results of the structural model are shown in Figure 2. In order to simplify the model, this chart only presents the related paths of the independent variable, mediating variable, and dependent variable, while the paths of control variables are omitted. Results revealed that professionalism was significantly positively related to job satisfaction (*β* = 0.75, *p* < 0.001), and employee job satisfaction had a significantly negative impact on turnover intention (*β* = −0.58, *p* < 0.001), after controlling for related demographic variables. Further, 5000 bootstrap samples from the raw dataset were generated to examine the indirect effect. The analysis results illustrated that the indirect influence of professionalism on turnover intention through job satisfaction was −0.44 (SE = 0.06, CI = [−0.54, −0.35], *p* < 0.000). The confidence interval of 95% did not include zero, indicating that social workers’ professionalism has a remarkable indirect influence on turnover intention through job satisfaction. However, the direct effect of professionalism on turnover intention was not significant, indicating that job satisfaction is a full mediation of the relationship between professionalism and turnover intention [79,80]. Therefore, Hypothesis 2 was supported, while Hypothesis 1 was not. The results support the theoretical model that promoting the level of worker professionalism has a significant indirect effect on the intention to leave the job through job satisfaction.

### 4.5. Test of Moderating Mediation Model

We used the technique of hierarchical regression via SPSS 24.0 to test the potential moderation of managerialism on the relationship between professionalism and job satisfaction, and professionalism and turnover intention, respectively. To reduce the potential effects of multicollinearity, all variables were standardized before conducting the regression [81]. Within the regression testing, each variable was created as a summated index. The technique of least squares was used with the control variables entered as a block in model 1 (age, education, and years of work experience), followed by the main effects in model 2 (professionalism and managerialism), and the interaction term in model 3. The results of hierarchical regression analyses are shown in Table 3. Interaction between professionalism and managerialism (Professionalism * managerialism) was significantly positively associated with job satisfaction (*β* = 0.112, *p* < 0.001), indicating that managerialism had a moderating effect on professionalism’s role in job satisfaction. Moreover, the results showed that the latent interaction variable, “professionalism * managerialism”, had a significant path coefficient (*β* = −0.121, *p* < 0.001), indicating that managerialism plays a moderating role in professionalism’s effect on employee turnover intention.

**Figure 3:**
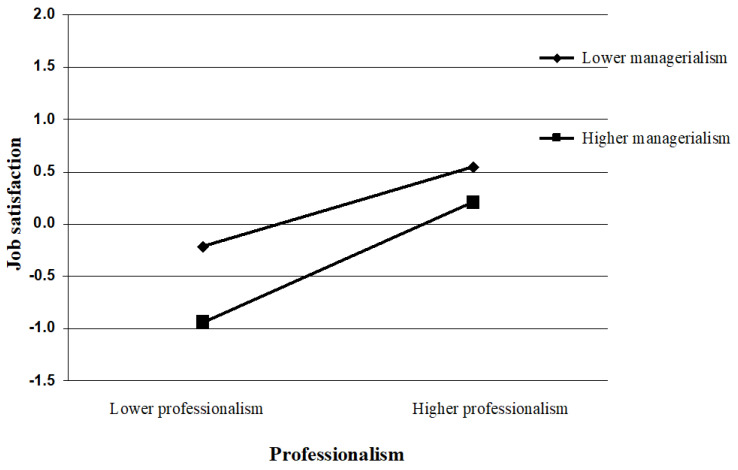
Moderating role of managerialism in the relationship between professionalism and job satisfaction.

**Figure 4:**
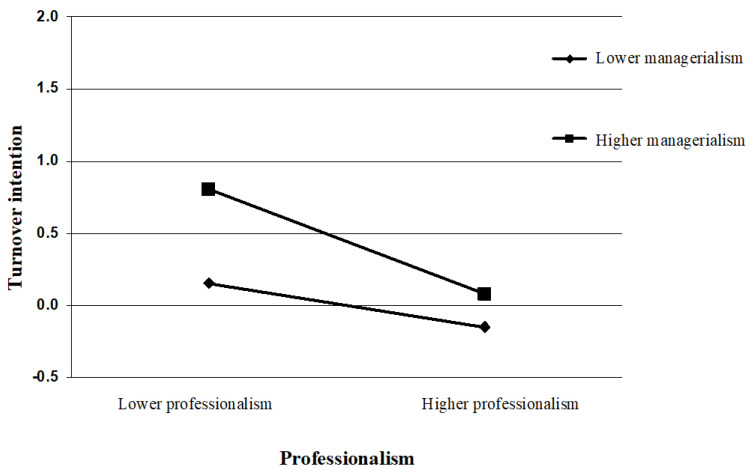
Moderating role of managerialism in the relationship between professionalism and turnover intention.

To further illustrate the moderating effects of managerialism, the study conducted simple slope analyses (see Figure 3 and Figure 4). The current study followed the procedures outlined by Aiken et al. [82] and plotted the relationship under high (1 *SD* above the mean) and low (1 *SD* below the mean) levels of innovative behavior. As shown in Figure 3, professionalism has a significant positive effect on job satisfaction (*β* = 0.38, *t* = 8.52, *p* < 0.001) with a lower managerialism level (M − 1SD). With a higher managerialism level (M + 1SD), professionalism still has a significant positive effect on job satisfaction (*β* = 0.58, *t* = 12.86, *p* < 0.001), and its prediction effect became even stronger. This indicated that professionalism’s effect on job satisfaction increased gradually as the employee’s perceived degree of managerialism increased. As can be seen in Figure 4, compared with the low-managerialism group (*β* = −0.15, *t* = −2.78, *p* < 0.01), higher levels of professionalism were more strongly predictive of lower levels of turnover intention among the high-managerialism group (*β* = −0.36, *t* = −6.61, *p* < 0.001). That is, a high level of managerialism may exacerbate the negative association between professionalism and turnover intention. Accordingly, Hypothesis 3a,b were not supported.

## 5. Discussion

Using the random sample of 667 social workers collected in the city of Guangzhou, Shenzhen, and Shanghai in mainland China, this study examines how social work practitioners’ turnover intention is associated with professionalism. Moreover, the moderating effect of managerialism was examined. First, the findings suggested that job satisfaction was a full mediation between a social worker’s professionalism and intention to leave the position. Second, a high level of managerialism exacerbates the positive relationship between professionalism and job satisfaction, as well as the negative association between professionalism and turnover intention. The findings supply empirical evidence supporting the proposed theoretical framework in this study.

### 5.1. Overall Model

The mediation model showed good indices of model fit, verifying the significant impact of professionalism on employees’ turnover intention. Further, the mediation model explained 38% of the variance for turnover intention, suggesting that professionalism and job satisfaction are crucial factors in explaining employees’ turnover intention. When social workers have a higher level of professionalism, they have stronger identification with and commitment to the profession, and thus they tend to feel more satisfied with their job, which in turn is related to a lower turnover intention. This is consistent with Herzberg’s theory [43,44], indicating that professional identification and commitment are positively associated with job satisfaction. Moreover, the findings echo many previous studies that demonstrate worker professionalism is positively related to job satisfaction [9,27,34]. However, the findings of these studies have mainly focused on the impact of professional identification and commitment, as a facet of professionalism on job satisfaction. Professionalism in the current study was conceptualized beyond identification and commitment. This study considered professionalism as a comprehensive measure that captured a variety of attitudinal attributes of social workers, referring to the value orientation and standards of practicing social workers, reflecting work attitudes and concepts, and incorporating attitudes representing levels of identification with and commitment to a particular profession. Furthermore, the findings of the current study were consistent with many previous studies on employee turnover that found job satisfaction was a critical and consistent predictor of turnover intention and actual turnover [21,83,84]. Therefore, the results of the present study supported the proposed theoretical framework suggesting that employees’ professionalism had an indirect link with turnover intention through job satisfaction.

In addition, job satisfaction was found to be a full mediation of the association between professionalism and turnover intention. Accordingly, there is no other way that an employee’s professionalism can affect his/her turnover intention without the process of individual satisfaction in the workplace. This is in line with previous literature that shows that personal and organizational variables affect turnover intention indirectly by affecting an employee’s level of job satisfaction [21,33]. Professionalism, as a type of professional attitude, affects employees’ work attitudes (job satisfaction), which in turn affects an individual’s behavioral intention and actual behavior [39]. This research finding sheds a light on the understanding of social work practitioners’ leaving process.

### 5.2. The Moderating Effect of Managerialism

Managerialism describes an ideology that business-like management practices should be adopted, the goal of which is to achieve efficiency and effectiveness through a configuration of ideas and practices [85]. However, the role of managerialism is a controversial issue that has elicited diverging opinions among researchers. Some argue that a strong focus on strategy, management, and for-profit practices dilutes an organization’s true purpose [53,86,87], while others find that applying for-profit practices, such as more pronounced market orientation, has a positive effect on organizational performance [88,89]. The results of the current study revealed that managerialism exacerbated the positive relationship between professionalism and job satisfaction, as well as the negative association between professionalism and turnover intention. In other words, managerialism strengthened the role of professionalism in promoting job satisfaction and in preventing employee turnover. Smeenk et al. [57] suggested that human service workers could be as committed as they were under a managerial regime but the effect of this commitment has changed as a result of increased managerialism. However, they did not find a moderating effect of managerialism on the relationship between organizational commitment and quality of job performance. The present study confirmed the change in the effect of organizational commitment as a result of increased managerialism, but the effect was on job satisfaction and turnover intention rather than on the quality of job performance. The findings are consistent with the thesis of considering managerialism as a positive role [51,58]. Smeenk and colleagues [57] found that a managerialism contradiction was largely absent among European university employees. However, previous studies on the positive effect of managerialism focused on its role in affecting organizational performance through using resources more effectively and efficiently [90]. This study enriches the positive role of managerialism in affecting professional attitudes.

One plausible explanation draws from the soft bureaucracy thesis [91], which demonstrated that middle managers act as a filter between top management and front-line operators, trying to communicate managerial imperatives but also to preserve the operators’ professional autonomy. Accordingly, employees’ perceived levels of managerialism may differ based on the different bureaucratic levels of managers they mostly interact with. The direct leaders of front-line social workers are mainly middle managers or even lower levels, and thus rather than eroding employees’ professionalism, managerialism plays a role in strengthening their professionalism.

The second perspective that may provide an explanation for the results that point to the positive effect of managerialism on professionalism is the development of organizational professionalism in knowledge-based, service-sector work [18,52]. It may be that professional employee values can co-exist with managerial values. This idea was supported by research by others. For instance, Gewirtz et al. [92] suggested that professional values have not entirely disappeared but that some employees switch between two sets of values according to the context, although accepting the new emphasis on markets and competition is often difficult for them.

Thirdly, social work organizations retain their character by adapting the managerialism ideology and values [57,93]. This is congruent with the Translation Theory, indicating that once a new management vision enters an organizational discourse, an ongoing process of meaning transformation is activated in which all actors discuss, interpret, modify, and alter the core ideas of the new management fashion [94]. Prichard and Willmott [95] indicated that the reception of managerialism ethos was largely influenced by localized practices and existing discursive regimes. Christensen and Lægreid [96] suggested that managerial features are filtered, interpreted, and adjusted in accordance with national and organizational contexts. Moreover, in the context of China, professionalism is also characterized by indigenization features [97]. Therefore, perceived managerialism may play a role in promoting the effect of professionalism in the Chinese cultural context.

Finally, under the mass purchase of social services by government bodies, a specifical Chinese social work organizational element can be added, that is, managerialism is often weakened and downgraded by a sort of “party governance” [98], in which political parties tend to occupy the managerial roles, so that managerialism does not only aim at economic efficiency but also to a political efficiency. In addition, professionalism in China is also characterized by indigenized features in terms of its purposes. For instance, social work in China is embedded in the existing system to pursue development and aims not only at promoting well-being and social justice but also at maintaining social stability and promoting political efficiency [99]. Therefore, social workers’ perceived managerialism may share commonalities with their interpretation of professionalism.

### 5.3. Limitations and Implications

There are some limitations to this study that should be addressed. First, because the data used are cross-sectional, the conclusions cannot predict the causal relationship of variables in this study. Longitudinal data could be used to provide empirical support for causality among social workers’ level of professionalism, job satisfaction, and their turnover intention. Second, Hood’s [69] managerialism scale was used in this study, which measures social workers’ perceived managerialism of their organizations. The perception of managerialism may to a certain extent differ from the real managerial situation of social work organizations. Future studies are encouraged to collect data on managerialism from multiple respondents such as leaders or managers specifically, as well as clients. Finally, the measures of our study were self-reported by social workers through an online survey, which may lead to the potential for the occurrence of common method variance (CMV). Although the CFA results on the influence of CMV alleviates some of this concern, future research might benefit from other methodological precautions, such as collecting data from different sources.

Despite these limitations, the present study has contributed to the development of knowledge by providing evidence to support the claim that employees’ professionalism prevents turnover intention through the promotion of job satisfaction, and the moderating role of managerialism in strengthening professionalism’s positive effect on job satisfaction as well as its negative effect on turnover intention. Our findings shed a light on the understanding of employee turnover by introducing the proposed casual chain of professionalism—job satisfaction—turnover intention into the context of social service workers’ leaving process, with a focus on professional and work attitudes. It further highlights that social workers’ professional attitudes (professionalism) affect turnover intention through the influence on their work attitudes (e.g., job satisfaction).

In addition, this study supports the applicability and feasibility of the hybridity perspective in understanding the relationship between professionalism and managerialism, which provides an alternative view on interpreting managerialism in social service settings in the context of China. The concept of hybridity has challenged the image of unresolvable conflicts existing between professional and managerial logic and demonstrated that although the two logics are still distinct, they can exist side by side within a manageable conflict [100]. Evidence for a managerialism contradiction is thus largely absent among Chinese social work practitioners.

Results suggested an additional way to reduce social workers’ turnover intention. One way is to improve employees’ job satisfaction by improving their level of professionalism. Although there are various factors associated with employees’ job satisfaction, for social workers, who often stay under the condition of low pay, few promotion opportunities, unclear career development, and low public identity, professionalism is a crucial factor [27]. High levels of professionalism represent high levels of identification and normative and affective commitment rather than continuance commitment to the profession [10]. It plays an important role in improving employees’ job satisfaction and reducing their turnover intention. Therefore, social work organizations should pay attention to fostering an organizational climate and culture of trust in a professional association, belief in public service and self-regulation, a sense of calling to the field, and autonomy. Particularly in the context of China, where social workers are diverse in background and many of them have not been professionally trained in universities [32]. Therefore, onsite training that emphasizes professionalism is extremely vital in promoting social work practitioners’ identification with and commitment to the profession as well as professional values and standards. In addition, “some dose” of managerialism in the right proportion should be used by the time of improving professionalism, which can help strengthen the effect of professionalism in preventing turnover intention. Accordingly, management training that focuses on organizational professionalism should be provided to social work organization leaders and managers as well. For instance, mixed or hybrid models of professionalism can be introduced to social work organizations, that is, professional work becomes a matter of combining professional and managerial principles. Managers are supposed to be more professional or have strong professional loyalties and management approaches should meaningfully manage professional work, while professionals should be trained to be able to work in-between competing logics such as involvement in managing service quality and to have a general management logic so as to combine professional self-governance with a general management logic. Generally speaking, professional action should be positioned within well-managed and organized surroundings that both respect and restrain professional spaces. 

## 6. Conclusions

This study conceptualized professionalism as a comprehensive measure that captured employees’ identification with and commitment to the profession in the social service organization. The findings reveal that professionalism prevents turnover intention through a positive effect on job satisfaction. In the future, social work leaders, educators, and organizations can pay more attention to social workers’ professionalism. Moreover, the relationship between professionalism and job satisfaction, and professionalism and turnover intention are moderated by managerialism. Specifically, the employee’s perceived level of managerialism strengthens the effects of professionalism in promoting employees’ job satisfaction and in preventing their turnover intention. Future studies may explore the potential causes further.

## Figures and Tables

**Table 1:** Participant demographics (*n* = 667).

Characteristics		Frequency (*n*)	Percentage (%)
Gender	Male	128	19.2
	Female	539	80.8
Age	<30	419	62.8
	30–40	217	32.5
	40+	31	4.6
Education	HS/TS diploma	31	4.6
	JC/VC degree	262	39.3
	Bachelor’s degree or above	374	56.1
Years of work experience	<2 years	229	34.3
	2–5 years	282	42.3
	6–10 years	115	17.2
	>10 years	41	6.1

Note. TS School = Technical Secondary School; JC = Junior College; VC = Vocational College.

**Table 2:** Results of descriptive statistics and correlation analysis for each variable (*n* = 667).

Variables	*M*	*SD*	1	2	3	4	5	6	7
1. Age	1.42	0.58	1						
2. Education	2.51	0.59	−0.03	1					
3. Years of work experience	1.95	0.87	0.44 **	0.10 *	1				
4. Professionalism	4.39	0.79	0.10 **	−0.20 **	0.04	1			
5. Managerialism	2.22	0.67	−0.11 **	0.09 *	−0.03	−0.57 **	1		
6. Job satisfaction	3.82	0.64	0.14 **	−0.12 **	0.08 *	0.61 **	−0.55 **	1	
7. Turnover intention	2.87	0.90	−0.18 **	0.08 *	−0.08 *	−0.37 **	0.39 **	−0.53 **	1

Note. * *p* < 0.05, ** *p* < 0.01.

**Table 3:** Moderated mediating model test (*n* = 667).

	Outcome Variable: Job Satisfaction			Outcome Variable: Turnover Intention		
	Model 1	Model 2	Model 3	Model 1	Model 2	Model 3
Age	0.117 **	0.047	0.050	−0.174 ***	−0.128 **	−0.131 **
Education	−0.116 **	−0.005	−0.008	0.073	0.010	0.013
Years of experience	−0.040	0.034	0.032	−0.014	−0.011	−0.009
Professionalism		0.441 ***	0.478 ***		−0.217 ***	−0.257 ***
Managerialism		−0.289 ***	−0.265 ***		0.247 ***	0.221 ***
Professionalism × Managerialism			0.112 ***			−0.121 **
*R* ^2^	0.033	0.438	0.450	0.038	0.201	0.214
Adj. *R*^2^	0.028	0.434	0.445	0.034	0.195	0.207
Δ*R*^2^	0.033	0.405	0.012	0.038	0.162	0.014
F	7.473 ***	238.300 ***	13.949 ***	8.832 ***	67.172 ***	11.374 **

Note. Standardized regression coefficients are presented. ** *p* < 0.01; *** *p* < 0.001.

## Data Availability

Data used in this work can be obtained from the corresponding author upon request.
